# Associations of sodium intake with obesity, metabolic disorder, and albuminuria according to age

**DOI:** 10.1371/journal.pone.0188770

**Published:** 2017-12-15

**Authors:** Se Won Oh, Ho Seok Koo, Kum Hyun Han, Sang Youb Han, Ho Jun Chin

**Affiliations:** 1 Department of Internal Medicine, Korea University Anam Hospital, Korea University College of Medicine, Seoul, Korea; 2 Department of Internal Medicine, Seoul Paik Hospital, Inje University College of Medicine, Seoul, Korea; 3 Department of Internal Medicine, Ilsan Paik Hospital, Inje University College of Medicine, Goyang, Korea; 4 Department of Internal Medicine, Seoul National University Bundang Hospital, Seoul National University, Seongnam, Korea; The University of Tokyo, JAPAN

## Abstract

Sodium intake is associated with obesity and metabolic disorder in the general population. However, sodium intake is significantly reduced according to the decrease of energy intake in older adults although the prevalence of obesity is higher than younger adults. We evaluate the association of sodium excretion (UNa) with blood pressure, obesity, metabolic disorders, and albuminuria according to age. An observational study using data from the Korean National Health and Nutrition Examination Survey IV-V (2008–2011) was performed (N = 18,146). The 24 hour UNa was estimated from a single fasting urine sample.Participants aged≥75 years showed the highest risk for hypertension (HTN) in the highest quartile of UNa (1.769, 95% CI, 1.174–2.665), and the risks for HTN increased with advancing age. Obesity was not associated with UNa in participants aged≥75 years, and hypertriglyceridemia and body fat were not related to UNa in participants aged≥65 years, although these values were significantly associated with UNa in participants aged<65 years. Impaired fasting glucose (IFG) and insulin resistance (IR) were associated with UNa only in participants aged 20–39 years. The highest quartile of UNa showed a 3.777 fold increased risk for albuminuria in those aged 20–39 years (95% CI, 1.130–12.630), and a 1.885 fold increased risk (95% CI, 1.156–3.075) among participants aged 40–64 years. In participants aged≥65 years, albuminuria was not associated with UNa. In contrast with HTN, UNa was not associated with albuminuria, obesity, hypertriglyceridemia, IFG, and IR in older adults despite a strong association in younger adults.

## Introduction

High sodium intake is associated with increased risk of hypertension (HTN), cardiovascular disease (CVD), decreased renal function, and death [1–6]. Current guidelines and studies recommend sodium restriction especially in patients with CVD and chronic kidney disease (CKD) [7, 8].

However, recent large-scale studies reported a J- or U-shaped relationship between sodium intake and outcomes. The risks for cardiovascular and all-cause mortality were increased in participants with low sodium intake, as well as in participants with high sodium intake [3, 6, 9]. In addition, sodium intake was not associated with 10-year death or risk for CVD in older adults [10].

Sodium intake stimulates thirst and appetite, and subsequently increases energy intake and extracellular volume [11, 12]. Sodium intake is associated with obesity and metabolic syndrome in the general population [13]. However, the relationship between sodium intake and obesity could be different in the elderly population. The elderly population is vulnerable to malnutrition, and low sodium intake may be related to inadequate caloric intake and volume depletion [14]. In addition, the main contributor of obesity in older adults is not likely to be increased energy intake, but rather reduced energy expenditure [15, 16]. On the other hand, blood pressure (BP) was demonstrated to be significantly more sensitive to salt intake in the oldest adults than that in the young adults [17, 18]. An increase of BP was only seen in the oldest adults at the end of 3 days of high sodium intake [18]. However, studies about the effect of sodium intake on BP, obesity, and metabolic disorders in the elderly population are lacking. In this study, we evaluated the association of BP, obesity, hypertriglyceridemia, impaired fasting glucose (IFG), and insulin resistance (IR) with sodium excretion in participants stratified according to age. In addition, we assessed the association between sodium excretion and albuminuria, which is a surrogate marker of CVD.

## Materials and methods

### Participants

The Korea National Health and Nutrition Examination Survey (KNHANES) is a nationwide representative survey of the health and nutritional status of the civilian Korean population. We analysed data from the fourth (IV-2, 3, 2008–2009) and fifth (V-1, 2, 2010–2011) KNHANES. The selection criteria for the study population were previously reported [13]. We divided the 18, 146 enrolled participants into groups based on their age: 20–39, 40–64, 65–74, and ≥75 years. In each age group, we additionally divided the participants into gender-specific quartile groups according to sodium excretion values. Participants in the KNHANES signed an informed consent form and the survey was approved by the institutional review board of Centers for Disease Control and Prevention in Korea (IRB No. 2010-02CON-21-C). The study was conducted in accordance with the Declaration of Helsinki.

### Measurements

Four nurses of the survey team of the Korea Centers for Disease Control and Prevention were in charge of measuring blood pressure. The nurses measured blood pressure three times manually as part of the health screening by mercury sphygmomanometer device (Baumanometer® Wall Unit 33(0850) (Baum, US)). Laboratory investigations, urinary sodium excretion, and body fat percentages were calculated as per previously documented methods [13]. Blood and urine samples, after an 8-h fast, were collected year-round and immediately processed, refrigerated, and transported in cold storage to the central laboratory (NeoDin Medical Institute, Seoul, South Korea) for analysis within 24 hours. Urine albumin was measured by turbidimetric assay (Albumin, Roche, Germany) using Hitachi automatic analyzer 7600 (Hitachi, Japan). Urine albumin was measured only in participants who had an examination in 2011 (N = 5119). The estimated glomerular filtration rate (eGFR) was estimated by using the Chronic Kidney Disease Epidemiology Collaboration (CKD-EPI) equation [19]. The three BP readings were obtained using a mercury sphygmomanometer, and the final BP value for individual participants was reported by calculating the mean of the second and third reading. IR assessment by the homeostatic assay (HOMA-IR) was calculated using the formula: HOMA-IR = [fasting glucose × fasting insulin/405] [20]. Levels of HOMA-IR were measured in 12,913 participants. Body fat percentage was determined by using dual-energy X-ray absorptiometry and measured in 14,370 participants.

### Definitions

The Kawasaki formula was used to estimate 24-hour urinary excretion of sodium using a single fasting urine sample and we applied these estimates as surrogates for sodium intake [21, 22]. HTN was defined as the presence of either (i) systolic BP (SBP) ≥140 mmHg or diastolic BP (DBP) ≥90 mmHg or (ii) if the patient was on antihypertensive medication at the time of interview. Participants were considered to have diabetes mellitus (DM) if they fulfilled at least one of the following 4 criteria: (i) fasting blood glucose ≥126 mg/dL; (ii) use of medication to decrease blood glucose level at the time of interview; (iii) use of insulin therapy at the time of interview; and (iv) self-report of having received a physician’s diagnosis of diabetes. Body mass index (BMI) was calculated on the basis of weight and height (kg/m^2^). Obesity was defined as a BMI ≥25 kg/m^2^. Abdominal obesity was defined as a waist circumference ≥ 90 cm in men and ≥ 80 cm in women based on the Asian criteria of abdominal obesity in International Diabetes Federation consensus [23]. Hypertriglyceridemia was defined as a serum triglyceride ≥ 150 mg/dL and IFG was defined as a fasting glucose ≥ 110 mg/dL. Participants whose HOMA-IR was in the top decile of the study population were defined as having IR [20]. Albuminuria was defined as a urine albumin creatinine ratio ≥ 30 mg/g·creatinine. Malignancy was defined as self-reported history of stomach, colon, liver, uterine cervix, breast, or other types of cancer. Myocardial infarction (MI), angina, and stroke were also defined by self-reported history. Current smoking was defined as smoking on ≥1 day within the previous month. Alcohol consumption was defined as drinking ≥2 alcoholic beverages in a month within the previous year.

### Statistical analysis

All analyses were performed using SPSS software (SPSS version 20.0, Chicago, IL, USA). Data are presented as the mean ± standard deviation for continuous variables and as a percentage for categorical variables. Differences were analysed using the chi-square test for categorical variables and analysis of variance for continuous variables. We analysed independent factors related to estimated sodium excretion by using multiple linear regression analysis. Analysis of covariance was used to adjust independent factors related to estimated sodium excretion. The risks and 95% confidence intervals (95% CIs) of factors associated with HTN, obesity, abdominal obesity, hypertriglyceridemia, IFG, IR, and albuminuria were calculated by using logistic regression analysis. A P value < 0.05 was considered statistically significant.

## Results

### Clinical characteristics

The clinical characteristics of the study participants are shown in Table 1. The medians of sodium excretion were 3.85 [3.04–4.83], 4.23[3.38–5.20], 4.33[3.47–5.30], and 4.11[3.21–5.04] in age group 20–39, 40–64, 65–74, and ≥75 years, respectively. The participants with older age tended to have a larger waist circumference, higher SBP, and higher aspartate aminotransferase (AST), alkaline phosphatase (ALP), glucose, cholesterol, and triglyceride levels (P < 0.001). In addition, there was a higher prevalence of DM, HTN, MI, angina, stroke and malignancy among older participants (P < 0.001) (Table 1). Estimated means of sodium excretion were the lowest in participants aged ≥75 years (3.839, 95% CI 3.712–3.966), and sodium excretion was 4.288 (95% CI 4.215–4.360), 4.370 (95% CI 4.340–4.399), and 4.204 (95% CI 4.123–4.286) in participants aged 20–39, 40–64, and 65–74 years, respectively after adjusting for age, SBP, BMI, glucose, haemoglobin, triglyceride, white blood cell count, high density lipoprotein, ALP, AST, alanine aminotransferase, cholesterol, eGFR, and energy intake. There were significant interactions between urinary sodium excretion and age on hypertension (*P*<0.001), obesity (*P* = 0.016), abdominal obesity (*P* = 0.008), hypertriglycemia (*P* = 0.013), impaired fasting glucose (*P*<0.001), and insulin resistance (*P* = 0.002).

**Table 1:** Clinical characteristics of study population.

	Age (years)				
	20–39 (N = 5671)	40–64 (N = 8813)	65–74 (N = 2700)	≥75 (N = 962)	*P*
Age (years)	31.2±5.5	51.7±7.1	69.3±2.8	78.3±3.2	<0.001
BMI (kg/m^2^)	23.0±3.6	24.1±3.1	24.0±3.2	22.9±3.3	<0.001
Waist circumference (cm)	78.1±10.5	82.5±9.2	84.3±9.1	81.9±9.8	<0.001
Men (%)	2672 (47.1)	3995 (45.3)	1194 (44.2)	409 (42.5)	0.010
SBP (mmHg)	110.4±12.5	121.3±16.7	130.4±17.2	133.6±17.8	<0.001
DBP (mmHg)	74.0±10.4	79.5±10.6	77.2±9.5	74.9±10.0	<0.001
eGFR (mL/min/1.73 m^2^)	109.2±12.4	94.3±11.6	83.3±9.6	77.5±8.8	<0.001
Hb (g/dL)	14.1±1.7	14.1±1.6	13.8±1.4	13.4±1.4	<0.001
WBC (1000/μL)	6.2±1.7	6.0±1.7	6.1±1.7	6.2±1.7	<0.001
AST (IU/L)	20.3±12.2	23.5±13.3	24.4±12.3	23.3±9.5	<0.001
ALT (IU/L)	21.2±21.4	22.9±16.3	20.7±12.6	18.0±10.6	<0.001
ALP (IU/L)	207.3±69.2	228.5±70.7	252.0±77.1	261.7±85.0	<0.001
Glucose (mg/dL)	90.3±14.3	99.8±23.8	103.5±24.4	103.6±27.3	<0.001
Cholesterol(mg/dL)	179.4±33.3	195.2±36.4	191.9±37.4	190.5±34.6	<0.001
TG (mg/dL)	116.1±105.2	144.6±120.2	143.8±97.7	131.0±76.0	<0.001
HDL (mg/dL)	54.8±12.9	52.4±12.7	50.2±12.0	49.9±12.4	<0.001
HOMA-IR*	2.3±1.5	2.5±2.0	2.7±3.1	2.7±2.5	<0.001
DM (%)	95 (1.9)	868 (10.1)	532 (20.3)	179 (19.3)	<0.001
HTN (%)	482 (9.6)	3001 (34.3)	1578 (59.0)	610 (64.6)	<0.001
Anti-HTN medication (%)	39 (0.7)	1597 (18.3)	1190 (44.5)	444 (47.0)	<0.001
MI (%)	1 (0.0)	61 (0.7)	49 (1.8)	8 (0.8)	<0.001
Angina (%)	11 (0.2)	146 (1.7)	108 (4.0)	38 (4.0)	<0.001
Stroke (%)	6 (0.1)	130 (1.5)	123(4.6)	55 (5.8)	<0.001
Malignancy (%)	26 (0.5)	309 (3.5)	144 (5.4)	55 (5.8)	<0.001
Current smoker (%)	2073 (36.8)	2629 (30.0)	652 (24.3)	237 (25.1)	<0.001
Alcohol (%)	2971 (53.0)	3940 (45.2)	834 (31.2)	246 (26.1)	<0.001

Abbreviations: BMI (body mass index), systolic blood pressure (SBP), diastolic blood pressure (DBP), estimated glomerular filtration rate (eGFR), hemoglobin (Hb), white blood cell (WBC), aspartate aminotransferase (AST), alanine aminotransferase (ALT), alkaline phosphatase (ALP), triglyceride (TG), high density lipoprotein (HDL), diabetes mellitus (DM), hypertension (HTN), and myocardial infarction (MI).

*Levels of HOMA-IR were measured in 12,913 participants.

### Association between SBP and sodium excretion according to age

**Fig 1:**
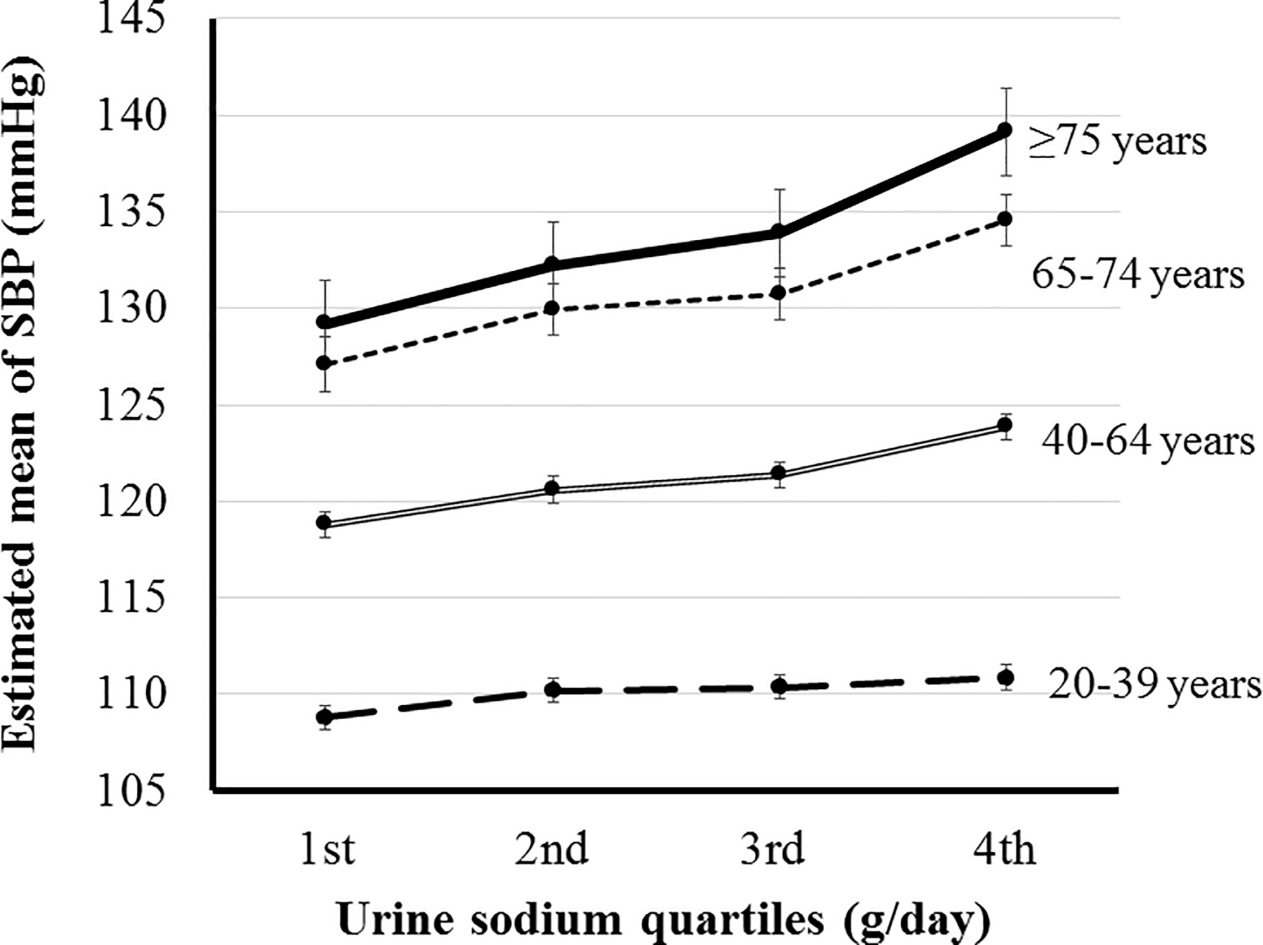
Estimated mean of systolic blood pressure (SBP) according to the sodium excretion in each age group. The SBP of participants in the highest quartile of sodium excretion was significantly higher than that of participants in the lowest, second, and third quartiles in all age groups except among those aged 20–39 years (*P*≤0.002). In the 20–39 years group, the SBP of the highest quartile of sodium excretion was only significantly higher than the lowest quartile (*P*<0.001). SBP was adjusted by age, body mass index, glucose, hemoglobin, white blood cell count, estimated glomerular filtration rate, triglyceride, high density lipoprotein, cholesterol, alkaline phosphatase, aspartate aminotransferase, alanine aminotransferase, and energy intake.

SBP was positively correlated with sodium excretion in all age groups (*P*<0.001). The differences in adjusted means of SBP were 2.058, 5.079, 7.426, and 9.920 mmHg between highest and lowest quartile of sodium excretion in age groups 20–39, 40–64, 65–74, and ≥75 years, respectively. The SBP of participants in the highest quartile of sodium excretion was significantly higher than that of participants in the lowest, second, and third quartiles in all age groups except among those aged 20–39 years (*P*≤0.002). In the 20–39 years group, the SBP of the highest quartile of sodium excretion was only significantly higher than the lowest quartile (*P*<0.001) (Fig 1).

**Fig 2:**
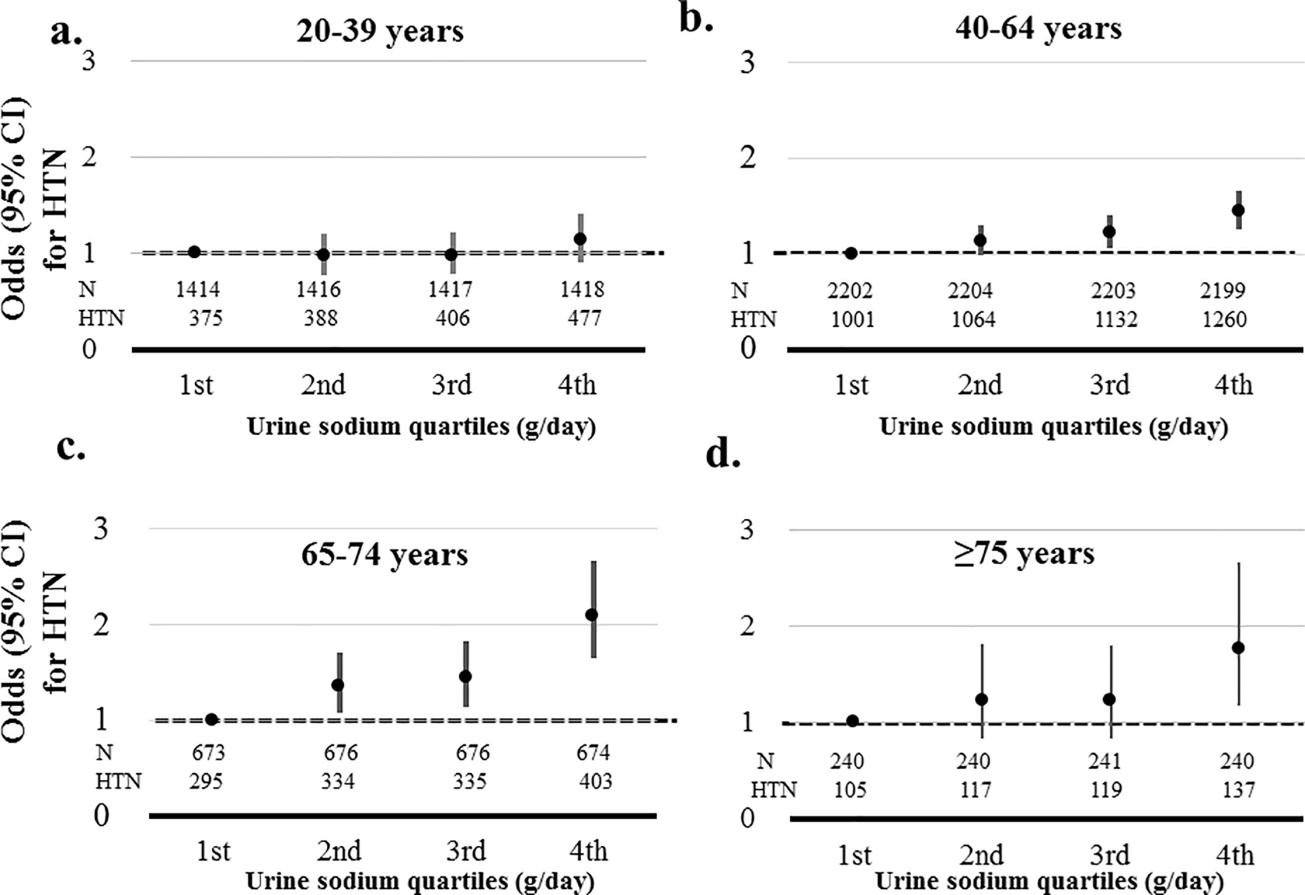
Association between hypertension (HTN) and sodium excretion quartiles according to age. Risks of HTN was adjusted by age, gender, body mass index, glucose, hemoglobin, estimated glomerular filtration rate, triglyceride, high density lipoprotein, aspartate aminotransferase, alanine aminotransferase, energy intake, diabetes mellitus, myocardial infarction, angina, stroke, malignancy, current smoker, and alcohol. a. Adjusted risks of HTN according to sodium excretion in age group 20–39 years. b. Adjusted risks of HTN according to sodium excretion in age group 40–64 years. c. Adjusted risks of HTN according to sodium excretion in age group 65–74 years. d. Adjusted risks of HTN according to sodium excretion in age group ≥ 75 years.

Participants aged ≥75 years showed the highest risk for HTN in the highest quartile of sodium excretion (1.769, 95% CI, 1.174–2.665) compared than the lowest quartile among age groups. The risks for HTN of participants in the highest quartile of sodium excretion increased according increasing age (1.128, 95% CI, 0.899–1.415; 1.438, 95% CI, 1.249–1.656; 2.097, 95% CI, 1.650–2.664; age 20–39 years, 40–64 years, and 65–74 years, respectively) (Fig 2).

### Association between obesity and sodium excretion according to age

**Fig 3:**
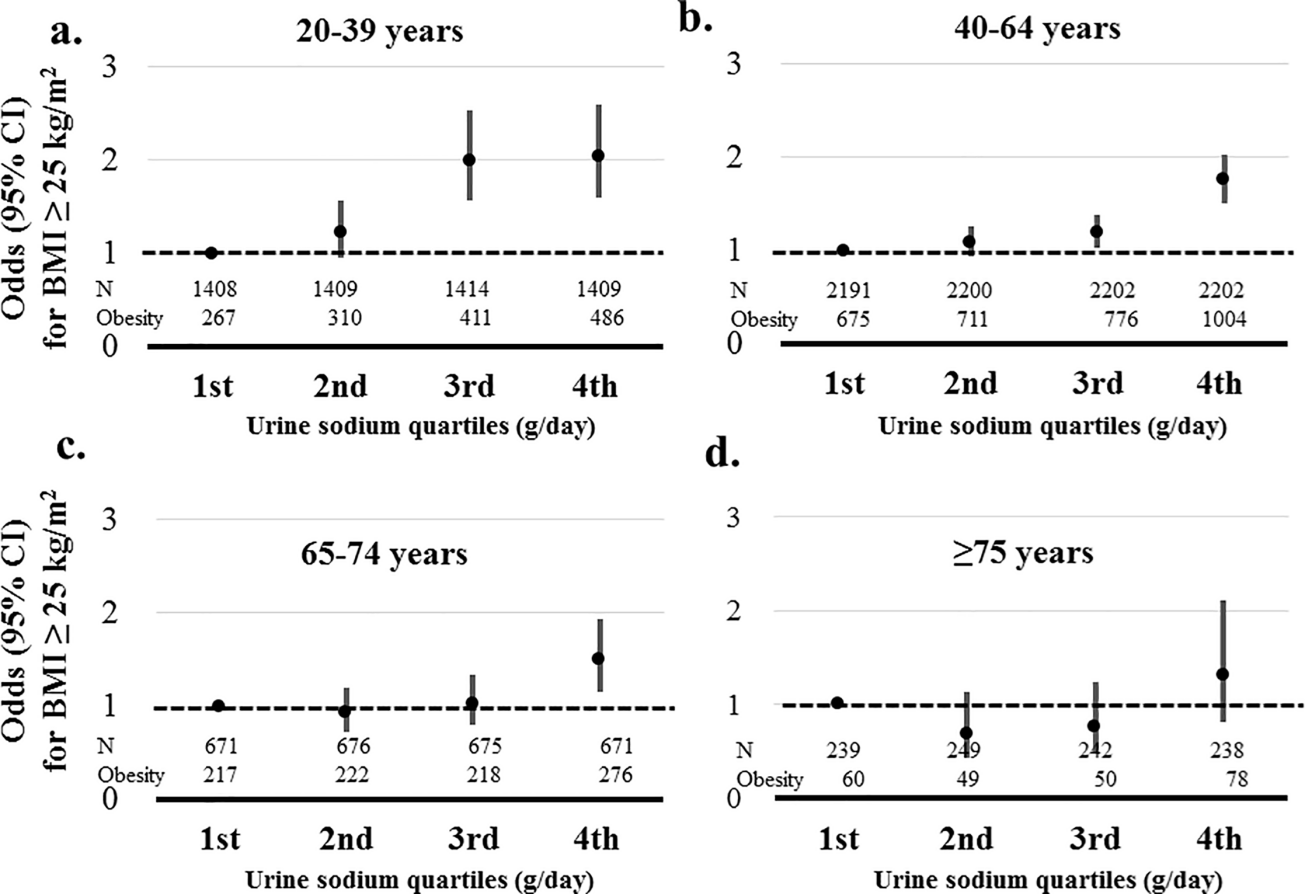
Association between obesity and sodium excretion quartiles according to age. Obesity was defined as a BMI ≥25 kg/m^2^. Risks of obesity was adjusted by age, gender, systolic blood pressure, glucose, hemoglobin, estimated glomerular filtration rate, triglyceride, high density lipoprotein, aspartate aminotransferase, alanine aminotransferase, energy intake, diabetes mellitus, myocardial infarction, angina, stroke, malignancy, current smoker, and alcohol. a. Adjusted risks of obesity according to sodium excretion in age group 20–39 years. b. Adjusted risks of obesity according to sodium excretion in age group 40–64 years. c. Adjusted risks of obesity according to sodium excretion in age group 65–74 years. d. Adjusted risks of obesity according to sodium excretion in age group ≥ 75 years.

Sodium excretion was significantly associated with obesity in participants with age 20–74 years (*P*≤0.002). However, sodium excretion was not associated with obesity in participants with age ≥75 years. Among participants in the highest sodium excretion quartile, the highest risk for obesity was observed in age group 20–39 years (2.038, 95% CI, 1.597–2.600) compared than the lowest quartile. The risks of obesity among participants in the highest sodium excretion quartile decreased according to increased age (1.756 (95% CI, 1.515–2.036); 1.497 (95% CI, 1.159–1.933); 1.307 (95% CI 0.810–2.110); age group 40–64, 65–74, and ≥75 years, respectively) compared than the lowest quartile of sodium excretion (Fig 3).

**Fig 4:**
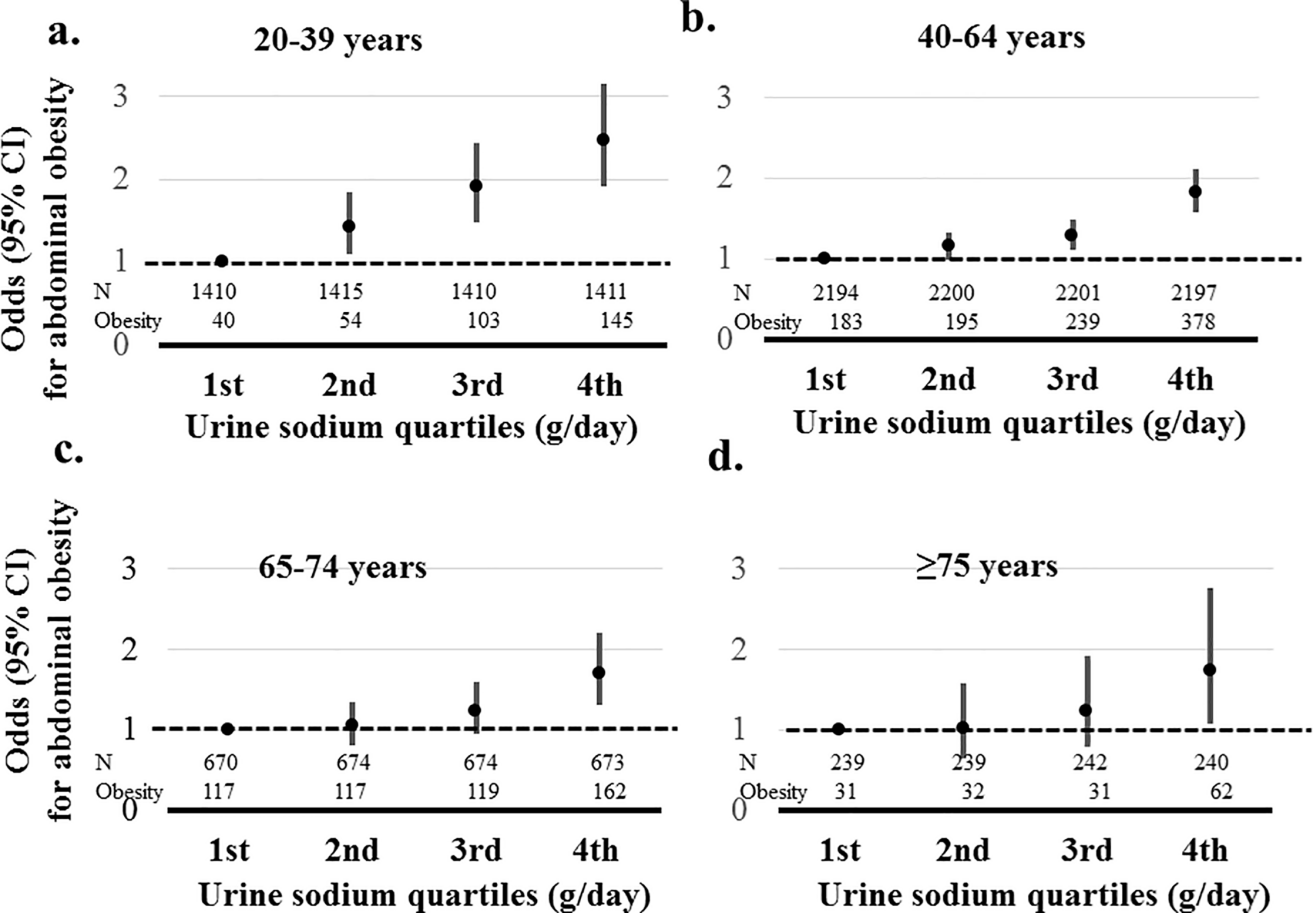
Association between abdominal obesity and sodium excretion quartiles according to age. Abdominal obesity was defined as a waist circumference ≥ 90 cm in men and ≥ 80 cm in women. Risks of abdominal obesity was adjusted by age, gender, systolic blood pressure, glucose, hemoglobin, estimated glomerular filtration rate, triglyceride, high density lipoprotein, aspartate aminotransferase, alanine aminotransferase, energy intake, diabetes mellitus, myocardial infarction, angina, stroke, malignancy, current smoker, and alcohol. a. Adjusted risks of abdominal obesity according to sodium excretion in age group 20–39 years. b. Adjusted risks of abdominal obesity according to sodium excretion in age group 40–64 years. c. Adjusted risks of abdominal obesity according to sodium excretion in age group 65–74 years. d. Adjusted risks of abdominal obesity according to sodium excretion in age group ≥ 75 years.

Sodium excretion was related to abdominal obesity in all age groups (*P*≤0.022). Similarly, the risks for abdominal obesity in the highest sodium excretion quartile were reduced according to increasing age (2.461 (95% CI, 1.919–3.156); 1.829 (95% CI, 1.575–2.122); 1.701 (95% CI, 1.305–2.216); 1.728 (95% CI, 1.082–2.762), age group 20–39, 40–64, 65–74, and ≥75 years, respectively) compared than the lowest quartile of sodium excretion (Fig 4).

### Association between sodium excretion and hypertriglyceridemia, impaired fasting glucose (IFG), insulin resistance (IR), and body fat percent according to age group

**Fig 5:**
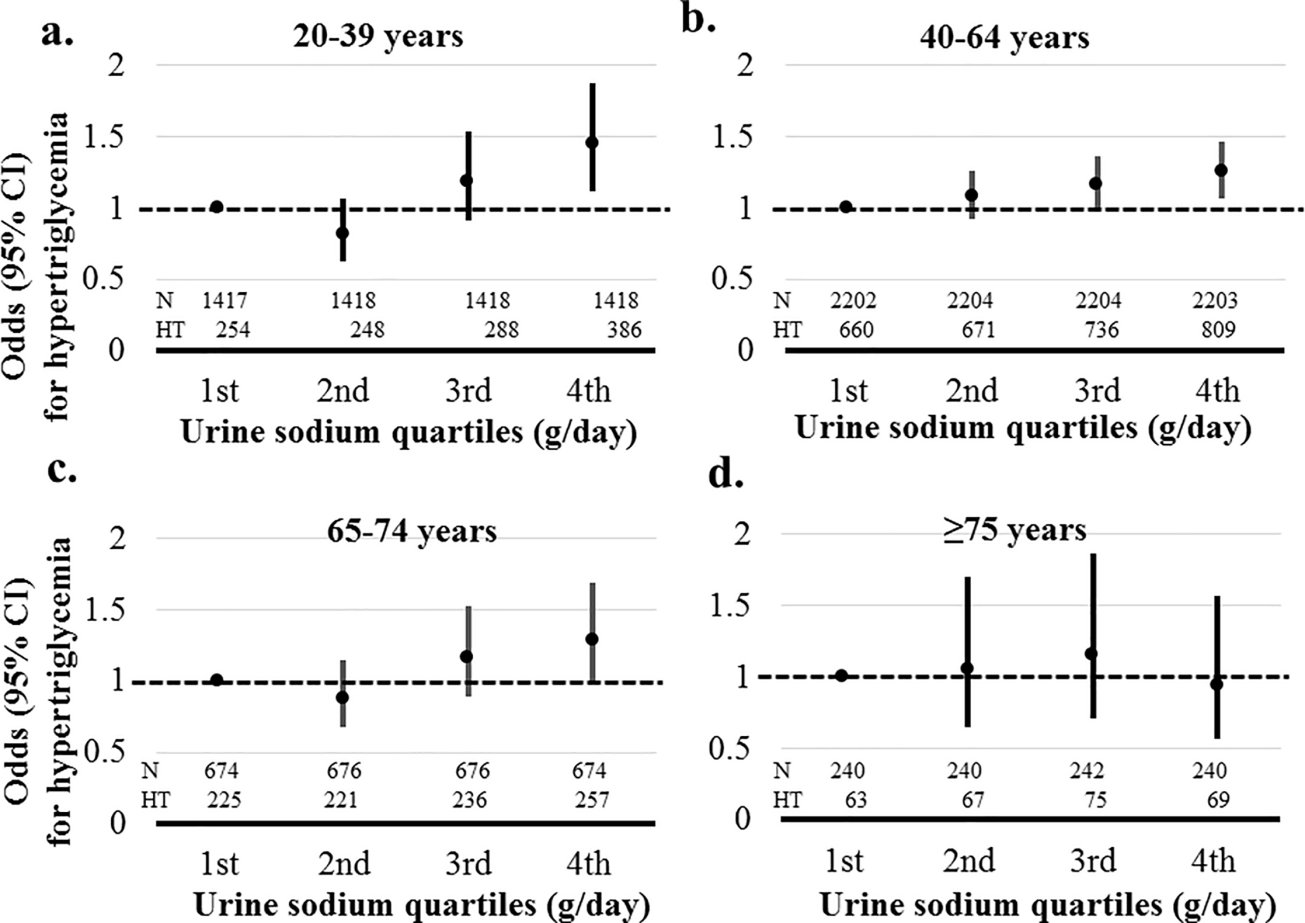
Association between sodium excretion quartiles and hypertriglycemia according to age. Hypertriglyceridemia was defined as a serum triglyceride ≥ 150 mg/dL. Risks of hypertriglyceridemia was adjusted by gender, systolic blood pressure, body mass index, glucose, hemoglobin, estimated glomerular filtration rate, high density lipoprotein, aspartate aminotransferase, alanine aminotransferase, energy intake, diabetes mellitus, myocardial infarction, angina, stroke, malignancy, current smoker, and alcohol. a. Adjusted risks of hypertriglycemia according to sodium excretion in age group 20–39 years. b. Adjusted risks of hypertriglycemia according to sodium excretion in age group 40–64 years. c. Adjusted risks of hypertriglycemia according to sodium excretion in age group 65–74 years. d. Adjusted risks of hypertriglycemia according to sodium excretion in age group ≥ 75 years.

Hypertriglyceridemia was significantly associated with sodium excretion in age groups 20–39 and 40–64 years (*P* = 0.006). Risks for hypertriglyceridemia in the highest sodium excretion quartile compared than the lowest quartile were 1.447 (95% CI, 1.115–1.880) and 1.252 (95% CI, 1.067–1.470) in age groups 20–39 and 40–64 years, respectively. In participants with age ≥ 65 years, hypertriglyceridemia was not related to sodium excretion (Fig 5).

**Fig 6:**
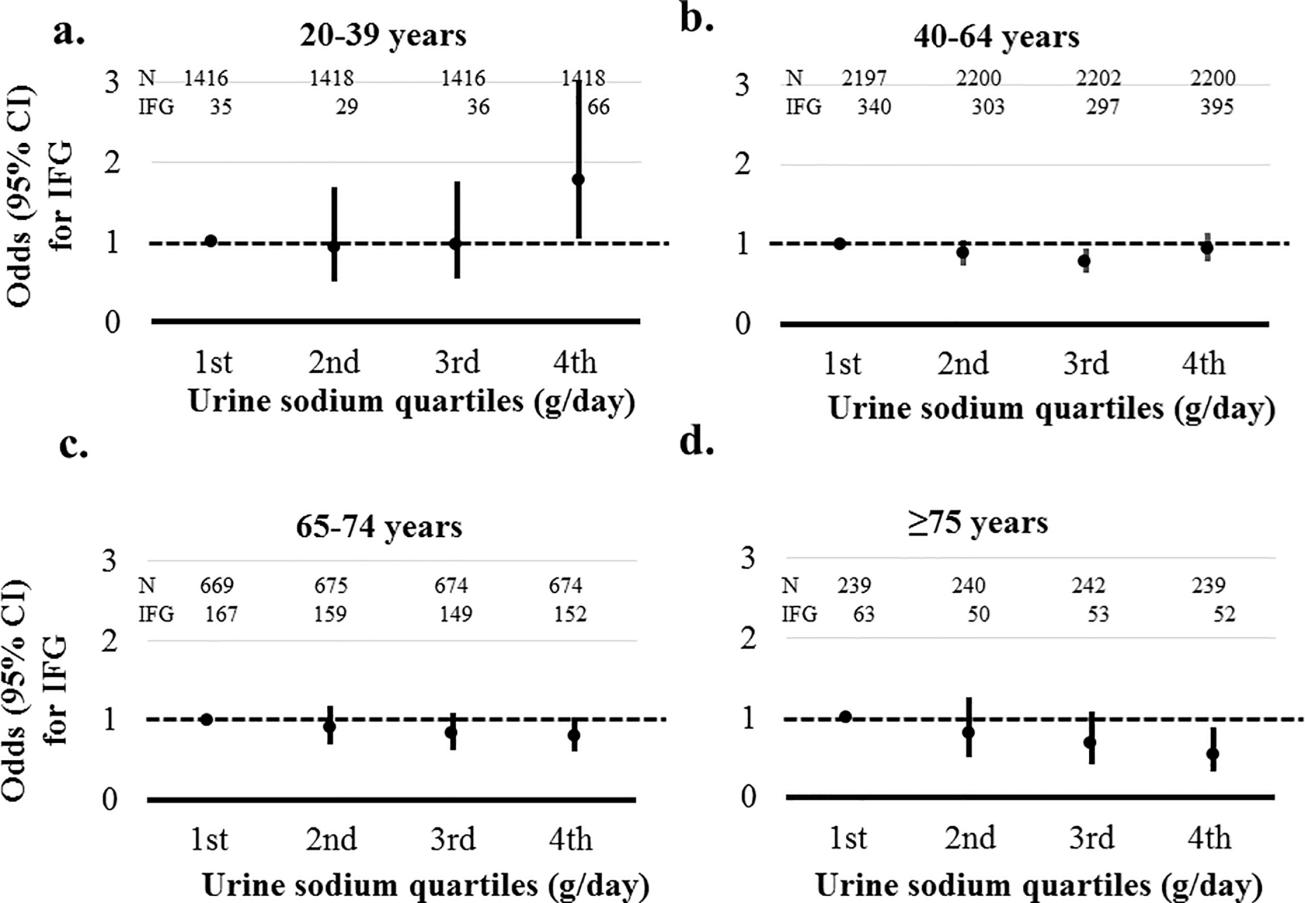
Association between sodium excretion quartiles and impaired fasting glucose (IFG) according to age. IFG was defined as a fasting glucose ≥ 110 mg/dL. Risks of IFG was adjusted by gender, systolic blood pressure, body mass index, hemoglobin, estimated glomerular filtration rate, high density lipoprotein, aspartate aminotransferase, alanine aminotransferase, energy intake, myocardial infarction, angina, stroke, malignancy, current smoker, and alcohol. a. Adjusted risks of IFG according to sodium excretion in age group 20–39 years. b. Adjusted risks of IFG according to sodium excretion in age group 40–64 years. c. Adjusted risks of IFG according to sodium excretion in age group 65–74 years. d. Adjusted risks of IFG according to sodium excretion in age group ≥ 75 years.

**Fig 7:**
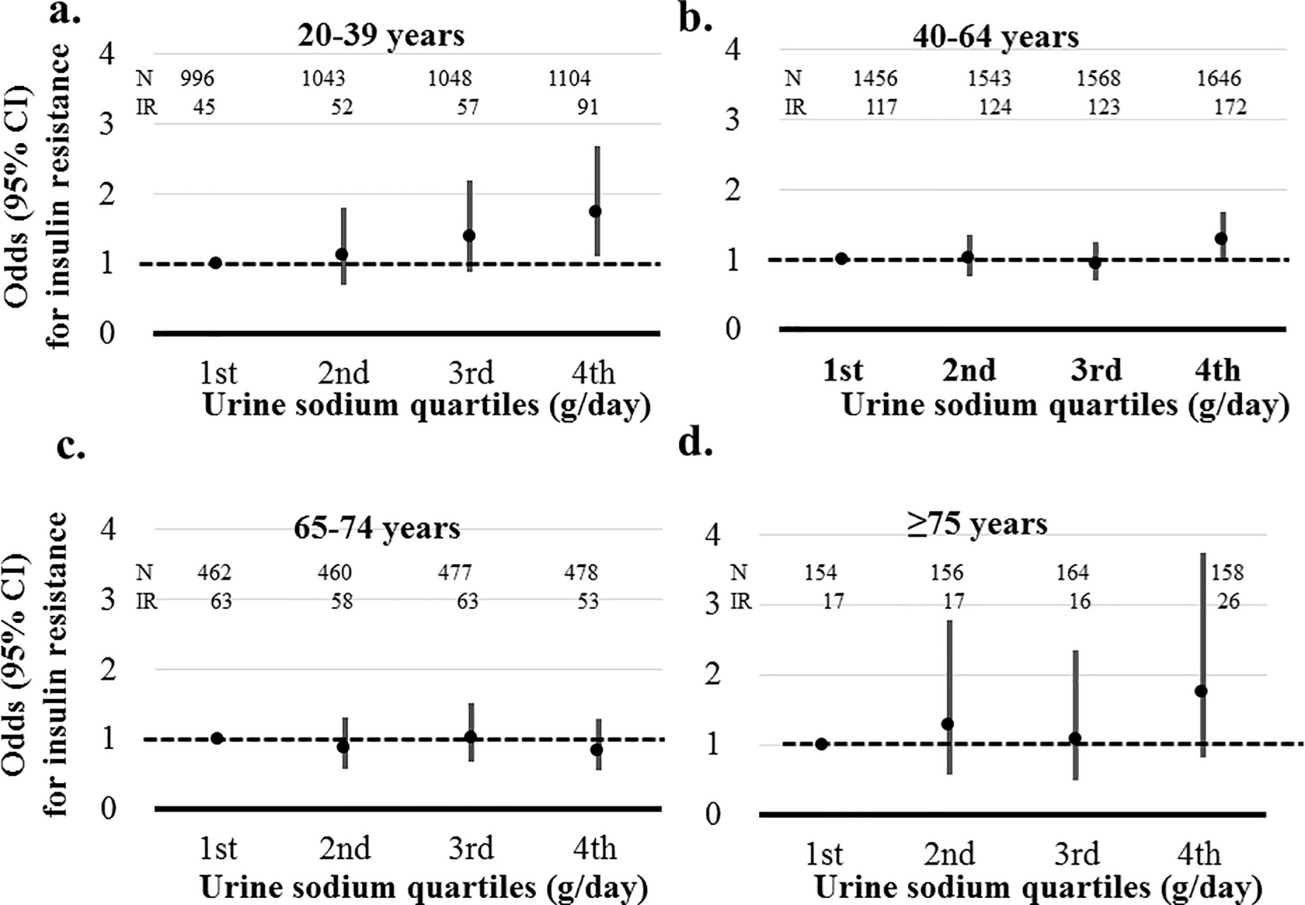
Association between sodium excretion quartiles and insulin resistance (IR) according to age. Participants whose HOMA-IR was in the top decile of the study population were defined as having IR. Risks of IR was adjusted by gender, hemoglobin, estimated glomerular filtration rate, aspartate aminotransferase, alanine aminotransferase, energy intake, hypertension, myocardial infarction, angina, stroke, malignancy, current smoker, and alcohol. a. Adjusted risks of IR according to sodium excretion in age group 20–39 years. b. Adjusted risks of IR according to sodium excretion in age group 40–64 years. c. Adjusted risks of IR according to sodium excretion in age group 65–74 years. d. Adjusted risks of IR according to sodium excretion in age group ≥ 75 years.

IFG and IR were associated with sodium excretion only in age group 20–39 years (*P*≤0.030). The highest quartile of sodium excretion showed a 1.775-fold increased risk of IFG (95% CI, 1.040–3.029) and 1.725-fold increased risk for IR (95% CI, 1.110–2.680) compared than the lowest quartile in participants with age 20–39 years. However, sodium excretion was not significantly associated with IFG and IR in other age groups (Figs 6 and 7).

**Fig 8:**
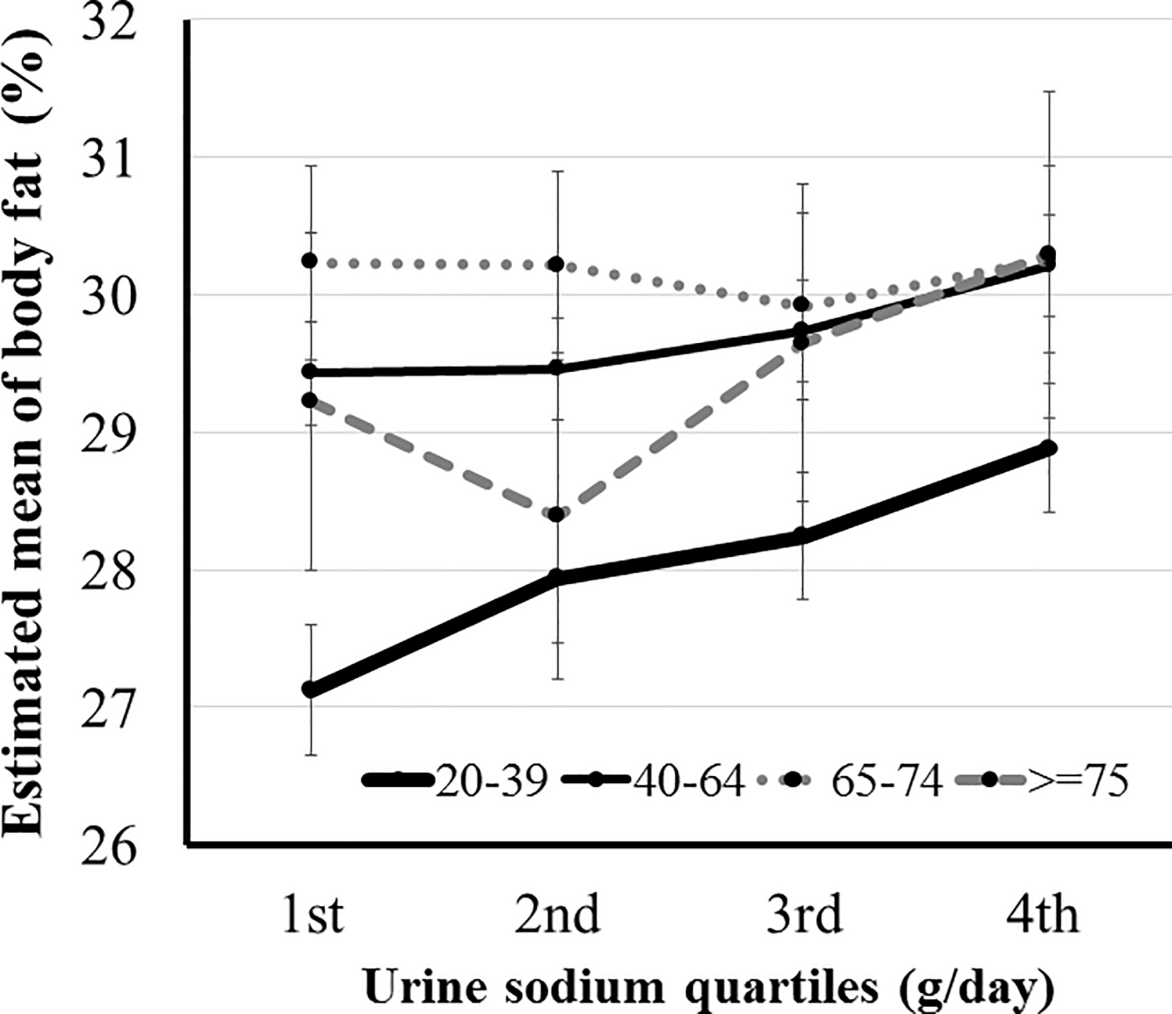
Association between sodium excretion quartile and body fat percent according to age. Body fat percent was positively associated with sodium excretion in age groups 20–39 and 40–64 years (*P*≤0.002). In participants with age ≥ 65 years, body fat percent was not related to sodium excretion. Body fat percent was adjusted by age, systolic blood pressure, glucose, white blood cell count, triglyceride, high density lipoprotein, cholesterol, alkaline phosphatase, aspartate aminotransferase, alanine aminotransferase, vitamin D and energy intake.

Body fat percent was positively associated with sodium excretion in age groups 20–39 and 40–64 years (*P*≤0.002). The body fat percent of participants in the highest quartile of sodium excretion was significantly higher than that of those in the lowest and second quartiles in age groups 20–39 and 40–64 years (*P*≤0.005). In participants with age ≥ 65 years, body fat percent was not related to sodium excretion (Fig 8).

### Association between sodium excretion and albuminuria according to age group

Sodium excretion was significantly associated with albuminuria in age groups 20–39 and 40–64 years (*P*≤0.019). The risks for albuminuria of the highest quartile of sodium excretion were reduced with increasing age, and albuminuria was not associated with sodium excretion in participants with age ≥ 65 years. The highest quartile of sodium excretion showed a 3.777 fold increase in the risk for albuminuria in age group 20–39 years (95% CI, 1.130–12.630), 1.885 (95% CI, 1.156–3.075) in age group 40–64 years, 1.317 (95% CI, 0.658–2.632) in 65–74 years, and 1.409 (95% CI, 0.487–4.080) in those aged ≥ 75 years compared than the lowest quartile (Table 2).

**Table 2:** Adjusted risks of albuminuria* according to sodium excretion in each age group.

	OR	95% CI	*P*		OR	95% CI	*P*
**20–39 years**				**40–64 years**			
**1**^**st**^		reference		**1**^**st**^		reference	
**2**^**nd**^	0.861	0.203–3.653	0.839	**2**^**nd**^	1.061	0.622–1.813	0.827
**3**^**rd**^	1.850	0.515–6.642	0.346	**3**^**rd**^	1.035	0.607–1.764	0.899
**4**^**th**^	3.777	1.130–12.630	0.031	**4**^**th**^	1.885	1.156–3.075	0.011
**65–74 years**				**≥75 years**			
**1**^**st**^		reference		**1**^**st**^		reference	
**2**^**nd**^	0.938	0.474–1.857	0.854	**2**^**nd**^	1.313	0.448–3.849	0.620
**3**^**rd**^	1.207	0.595–2.449	0.601	**3**^**rd**^	0.703	0.206–2.396	0.573
**4**^**th**^	1.317	0.658–2.632	0.437	**4**^**th**^	1.409	0.487–4.080	0.527

*Risks of albuminuria was adjusted by age, sex, body mass index, estimated glomerular filtration rate, hemoglobin, aspartate aminotransferase, alanine aminotransferase, high density lipoprotein, energy intake, diabetes, hypertension, smoking, alcohol, stroke, and myocardial infarction. Albuminuria was defined as a urine albumin creatinine ratio ≥ 30 mg/g·creatinine. Urine albumin was measured only in participants who had an examination in 2011 (N = 5119).

## Discussion

In this study, we demonstrated that compared to younger age groups, BP was most strongly associated with sodium excretion in the oldest group of participants. In contrast with BP, obesity was not associated with sodium excretion in participants with age ≥75 years. Hypertriglyceridemia and body fat percent were not related to sodium excretion in participants with age ≥65 years, although these factors were significantly associated with sodium excretion in younger adults. IFG and IR were associated with sodium excretion only in age group 20–39 years. Similarly, albuminuria was not significantly associated with sodium excretion in participants with age ≥65 years.

Compared to younger participants, the BP of elderly patient is more sensitive to salt intake [17, 18]. The ability of sodium excretion is reduced in the elderly because of a multitude of factors including a decrease in renal function, reduced synthesis of natriuretic substances, and reduced activity of membrane sodium/potassium-adenosine triphosphatase [17, 24, 25]. Salt taste acuity also reduces with advancing age, and increased sodium intake is observed in middle and old ages [13, 26]. However, total energy and sodium intake were significantly decreased in very elderly participants [13]. In our study, the risk of high SBP in the highest sodium excretion group was increased with advancing age, although total sodium excretion was lowest in participants aged ≥75 years. The increase in salt sensitivity with advancing age may not be due to increased salt intake, but rather to decreased capacity to excrete a salt load.

Sodium intake may be associated with an increase of thirst and appetite, and increased energy intake is an important cause of obesity. Sodium excretion was positively associated with obesity measures in the younger adults. However, we found a weak or no association of obesity and metabolic disorder with sodium excretion in older adults. Although energy intake does not change or even decrease with advancing age, obesity increases with aging [27]. Aging is related to a decline of energy expenditure as evidenced by changes in the resting metabolic rate, the thermic effect of food, and amount of physical activity [15, 16]. Obesity in the elderly is also related to changes the hormonal environment, which includes a decline of growth hormone and testosterone, decreased responsiveness to thyroid hormone and leptin, and increased prolactin and cortisol levels [15, 16, 28]. The main contributor to obesity in the elderly is reduced energy expenditure rather than increased energy intake. Therefore, sodium excretion was not associated with obesity measures in the elderly in this study.

Sodium excretion was significantly associated with albuminuria in participants aged < 65 years, but not in those aged ≥65 years. Albuminuria is a surrogate marker of endothelial damage in cardiovascular and renal disease [29]. Obesity, IFG, IR, and hypertriglyceridemia are potent risk factors for CVD; indeed, the fact that there was no association of these factors with sodium excretion in the elderly might have affected the association of albuminuria with sodium excretion in this group.

Many studies showed a J- or U-shaped association of sodium intake with cardiovascular and all-cause mortality [3, 6, 9]. Furthermore, no association was found between sodium intake and mortality in older adults [10]. These inconsistencies in results were explained on account of differences in study methodology, study population, variations of outcome measures, and study design [29]. Considering strong attributions to mortality of CVD risk factors, the study including old population might show no relationships between sodium intake and long-term outcomes.

The strength of this study is that we used a large and nationwide data set representative of the general population, and urine sodium excretion and other values were measured consistently among all participants with an identical method to measure urine sodium and other measures. Second, to the best of our knowledge, this is the first study to evaluate the association between obesity measures and sodium excretion according to age. A spot urine test is convenient in the clinical setting, and estimated sodium excretion using the Kawasaki formula correlated well with 24 hour urinary sodium excretion values in a previous study [22]. Diurnal variation in sodium excretion levels could be a limitation of estimated 24 hour urinary sodium excretion using spot urine specimens. However, we noted strong association between sodium excretion and sodium intake by questionnaire (P<0.001, B = 4.372; 95% CI, 4.332–4.411). Despite its strengths, this study also has some limitation. First, the study population comprised only Koreans, and race differences were not assessed in this study. Second, data was not adjusted for participants’ medication histories. KNHANES only provided whether the hypertensive participants administer anti-hypertensive medications or not, and there was no data about the kinds of anti-hypertensive medications. Sodium excretion could be affected by anti-hypertensive medication such as diuretics. Therefore, sodium excretion might be a less effective marker to determine sodium intake in elderly population who take anti-hypertensive medication. However, excluding the participants with anti-hypertensive medication (N = 3270) and nonresponse (N = 158), we re-analysed about the association of sodium excretion with blood pressure, albuminuria and metabolic abnormalities (N = 14,718). Similar results were notified (S1, S2 and S3 Fig and S1 Table). Finally, this was a cross-sectional study, and therefore causality cannot be assessed. In contrast with HTN, sodium excretion was not associated with obesity, IFG, IR, hypertriglyceridemia, and body fat percent in older adults. However, these CVD risk factors were significantly associated with sodium excretion in younger adults. Albuminuria, which is representative of cardiovascular damage, was associated with sodium excretion in younger, but not older adults. These findings suggest that sodium intake may differentially affect the risk of CVD in participants of different ages. Further studies are required to evaluate the association between sodium excretion and long-term outcomes in the elderly.

## Supporting information

S1 Fig Estimated mean of systolic blood pressure (SBP) according to the sodium excretion in each age group excluding participants with anti-hypertensive medication. The SBP of participants in the highest quartile of sodium excretion was significantly higher than that of participants in the lowest, second, and third quartiles in all age groups except among those aged 20–39 years (*P*≤0.027). In the 20–39 years group, the SBP of the highest quartile of sodium excretion was only significantly higher than the lowest quartile (*P*<0.001). SBP was adjusted by age, body mass index, glucose, hemoglobin, white blood cell count, estimated glomerular filtration rate, triglyceride, high density lipoprotein, cholesterol, alkaline phosphatase, aspartate aminotransferase, alanine aminotransferase, and energy intake.
(TIF)

S2 Fig Estimated mean of systolic blood pressure (SBP) according to the sodium excretion in each age group in participants with anti-hypertensive medication. The SBP of participants in the highest quartile of sodium excretion was significantly higher than that of participants in the lowest, second, and third quartiles in aged 40–64 years and ≥ 75 years (*P*≤0.021). In the 65–74 years group, the SBP of the highest quartile of sodium excretion was significantly higher than the lowest and second quartiles (*P*≤0.020). In the 20–39 years group, SBP was not significantly associated with sodium excretion. SBP was adjusted by age, body mass index, glucose, hemoglobin, white blood cell count, estimated glomerular filtration rate, triglyceride, high density lipoprotein, cholesterol, alkaline phosphatase, aspartate aminotransferase, alanine aminotransferase, and energy intake.
(TIF)

S3 Fig Association between sodium excretion quartiles and hypertriglycemia according to age excluding participants with anti-hypertensive medication. Hypertriglyceridemia was significantly associated with sodium excretion in age groups 20–39 and 40–64 years (*P*≤0.006). Risks for hypertriglyceridemia in the highest sodium excretion quartile compared than the lowest quartile were 1.442 (95% CI, 1.108–1.877) and 1.372 (95% CI, 1.138–1.655) in age groups 20–39 and 40–64 years, respectively. In participants with age ≥ 65 years, hypertriglyceridemia was not related to sodium excretion. Hypertriglyceridemia was defined as a serum triglyceride ≥ 150 mg/dL. Risks of hypertriglyceridemia was adjusted by gender, systolic blood pressure, body mass index, glucose, hemoglobin, estimated glomerular filtration rate, high density lipoprotein, aspartate aminotransferase, alanine aminotransferase, energy intake, diabetes mellitus, myocardial infarction, angina, stroke, malignancy, current smoker, and alcohol. **a. Adjusted risks of hypertriglycemia according to sodium excretion in age group 20–39 years. b. Adjusted risks of hypertriglycemia according to sodium excretion in age group 40–64 years. c. Adjusted risks of hypertriglycemia according to sodium excretion in age group 65–74 years. d. Adjusted risks of hypertriglycemia according to sodium excretion in age group ≥ 75 years**.
(TIF)

S1 Table Adjusted risks of albuminuria* according to sodium excretion in each age group excluding participants with anti-hypertensive medication. Sodium excretion was significantly associated with albuminuria in age groups 20–39 years excluding participants with anti-hypertensive medication (*P* = 0.033). The risks for albuminuria were not associated with sodium excretion in participants with age ≥ 40 years excluding participants with anti-hypertensive medication. Risks of albuminuria was adjusted by age, sex, body mass index, estimated glomerular filtration rate, hemoglobin, aspartate aminotransferase, alanine aminotransferase, high density lipoprotein, energy intake, diabetes, hypertension, smoking, alcohol, stroke, and myocardial infarction.
(DOCX)
