# Supplementary figures and images for: Associations of sodium intake with obesity, metabolic disorder, and albuminuria according to age
Source: PLoS One. 2017 Dec 15;12(12):e0188770. doi: 10.1371/journal.pone.0188770 (PMC5731764; doi:10.1371/journal.pone.0188770)

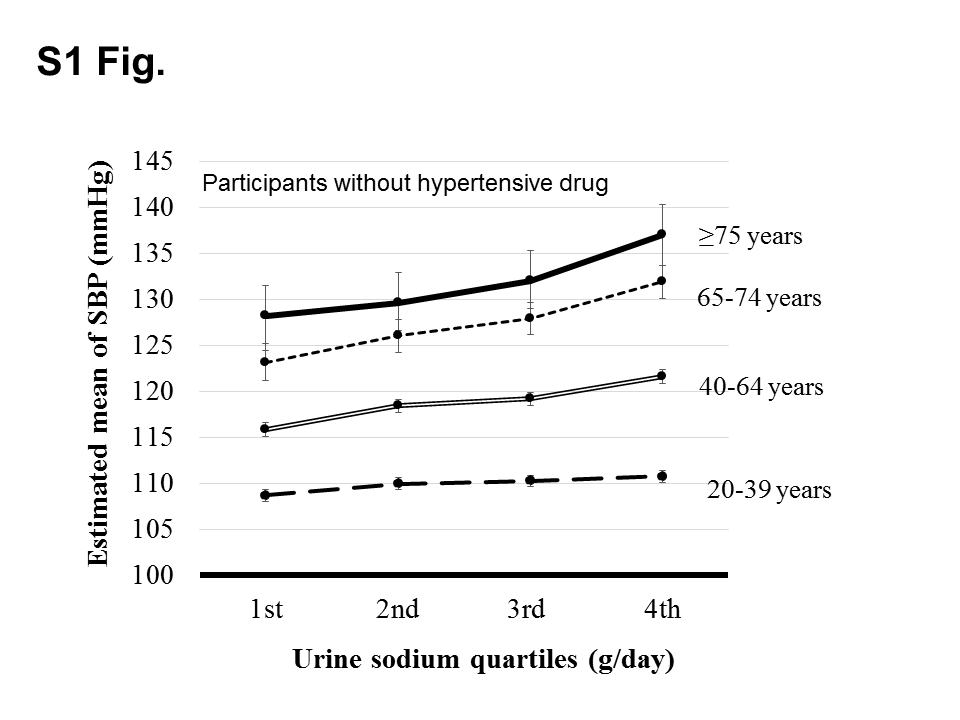

Supplement: S1 Fig — The SBP of participants in the highest quartile of sodium excretion was significantly higher than that of participants in the lowest, second, and third quartiles in all age groups except among those aged 20–39 years (P≤0.027). In the 20–39 years group, the SBP of the highest quartile of sodium excretion was only significantly higher than the lowest quartile (P<0.001). SBP was adjusted by age, body mass index, glucose, hemoglobin, white blood cell count, estimated glomerular filtration rate, triglyceride, high density lipoprotein, cholesterol, alkaline phosphatase, aspartate aminotransferase, alanine aminotransferase, and energy intake. (TIF) [file pone.0188770.s001.TIF]

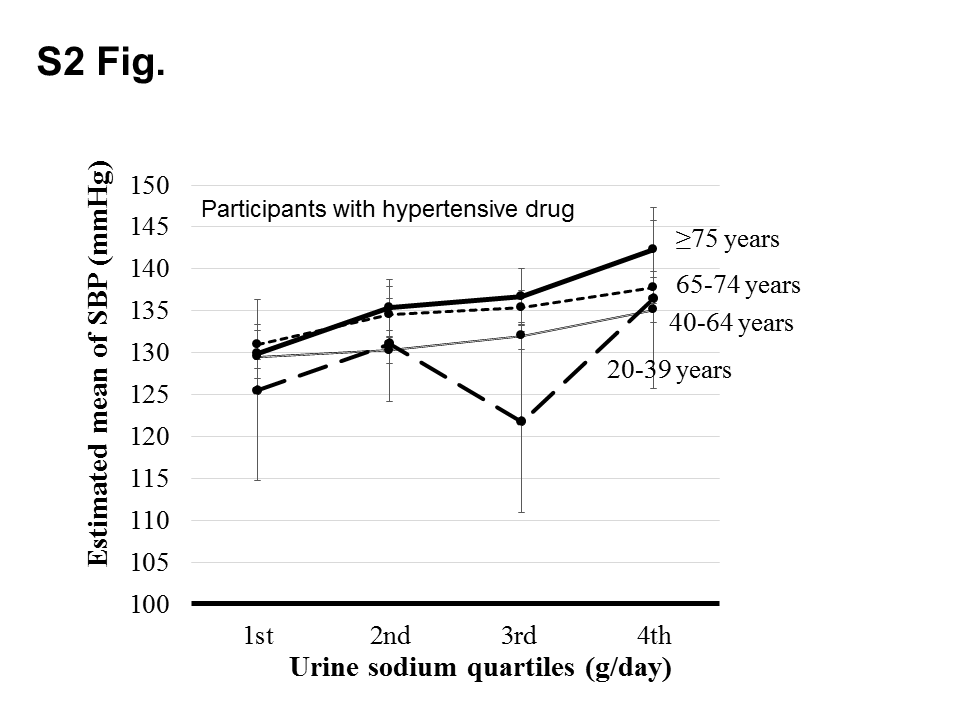

Supplement: S2 Fig — The SBP of participants in the highest quartile of sodium excretion was significantly higher than that of participants in the lowest, second, and third quartiles in aged 40–64 years and ≥ 75 years (P≤0.021). In the 65–74 years group, the SBP of the highest quartile of sodium excretion was significantly higher than the lowest and second quartiles (P≤0.020). In the 20–39 years group, SBP was not significantly associated with sodium excretion. SBP was adjusted by age, body mass index, glucose, hemoglobin, white blood cell count, estimated glomerular filtration rate, triglyceride, high density lipoprotein, cholesterol, alkaline phosphatase, aspartate aminotransferase, alanine aminotransferase, and energy intake. (TIF) [file pone.0188770.s002.TIF]

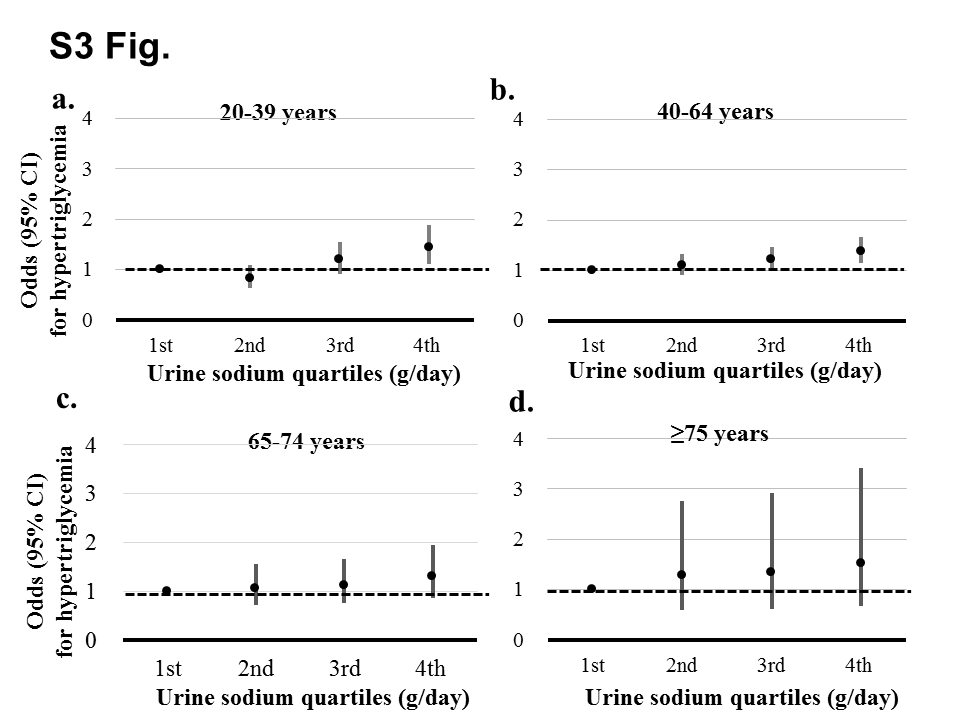

Supplement: S3 Fig — Hypertriglyceridemia was significantly associated with sodium excretion in age groups 20–39 and 40–64 years (P≤0.006). Risks for hypertriglyceridemia in the highest sodium excretion quartile compared than the lowest quartile were 1.442 (95% CI, 1.108–1.877) and 1.372 (95% CI, 1.138–1.655) in age groups 20–39 and 40–64 years, respectively. In participants with age ≥ 65 years, hypertriglyceridemia was not related to sodium excretion. Hypertriglyceridemia was defined as a serum triglyceride ≥ 150 mg/dL. Risks of hypertriglyceridemia was adjusted by gender, systolic blood pressure, body mass index, glucose, hemoglobin, estimated glomerular filtration rate, high density lipoprotein, aspartate aminotransferase, alanine aminotransferase, energy intake, diabetes mellitus, myocardial infarction, angina, stroke, malignancy, current smoker, and alcohol. a. Adjusted risks of hypertriglycemia according to sodium excretion in age group 20–39 years. b. Adjusted risks of hypertriglycemia according to sodium excretion in age group 40–64 years. c. Adjusted risks of hypertriglycemia according to sodium excretion in age group 65–74 years. d. Adjusted risks of hypertriglycemia according to sodium excretion in age group ≥ 75 years. (TIF) [file pone.0188770.s003.TIF]
